# Distinct binding pattern of EZH2 and JARID2 on RNAs and DNAs in hepatocellular carcinoma development

**DOI:** 10.3389/fonc.2022.904633

**Published:** 2022-12-09

**Authors:** Zhili Wen, Ke He, Meixiao Zhan, Yong Li, Fei Liu, Xu He, Yanli Wei, Wei Zhao, Yu Zhang, Yaqiang Xue, Yong Xia, Fenfen Wang, Zhenglin Xia, Yongjie Xin, Yeye Wu, Xiaopeng Duan, Jing Xiao, Feng Shen, Yuliang Feng, Guoan Xiang, Ligong Lu

**Affiliations:** ^1^ Department of Gastroenterology, Second Affiliated Hospital, Nanchang University, Nanchang, China; ^2^ Infectious Hospital, Nanchang University, Nanchang, China; ^3^ Department of General Surgery, Guangdong Second Provincial General Hospital, Guangzhou, China; ^4^ Department of Biochemistry, Zhongshan School of Medicine, Sun Yat-sen University, Guangzhou, China; ^5^ Center for Stem Cell Biology and Tissue Engineering, Key Laboratory of Ministry of Education, Sun Yat-sen University, Guangzhou, China; ^6^ Zhuhai Interventional Medical Center, Zhuhai Precision Medical Center, Zhuhai People’s Hospital, Zhuhai Hospital Affiliated with Jinan University, Jinan University, Zhuhai, China; ^7^ Center for Genome Analysis, ABLife Inc., Wuhan, China; ^8^ Laboratory of Human Health and Genome Regulation, ABLife Inc., Wuhan, China; ^9^ Department of Hepatic Surgery, The Eastern Hepatobiliary Surgery Hospital, Navy Medical University, Shanghai, China

**Keywords:** EZH2, JARID2, CLIP-seq, ChIP-seq, hepatocellular carcinoma

## Abstract

Hepatocellular carcinoma (HCC) is one of the most malignant cancers worldwide, with high mortality. However, the molecular regulatory mechanisms of liver cancer, especially transcriptional and post-transcriptional mechanisms, should be further studied. Here we used chromatin and cross-linking immunoprecipitation with high throughput sequencing methods (ChIP-seq and CLIP-seq) to capture the global binding profiles on RNAs and DNAs of Enhancer of zeste homolog 2 (EZH2) and its partner Jumonji And AT-Rich Interaction Domain Containing 2 (JARID2) in liver carcinoma cell lines (HepG2) and normal liver cell line (THLE-2), respectively. We also integrated HCC transcriptome data from the TCGA to analyze the expression pattern of bound genes. We found that EZH2 and JARID2 both showed distinct binding profiles between HepG2 and THLE-2 cells. By binding to the primary RNAs, bound transcripts of EZH2 and JARID2 in HepG2 showed significantly increased transcriptional levels in HCC patients. By performing gene set enrichment analysis (GSEA), the bound transcripts were also highly related to HCC development. We also found EZH2 and JARID2 could specifically bind to several long noncoding RNAs (lncRNAs), including H19. By exploring the DNA binding profile, we detected a dramatically repressed DNA binding ability of EZH2 in HepG2 cells. We also found that the EZH2-bound genes showed slightly increased transcriptional levels in HepG2 cells. Integrating analysis of the RNA and DNA binding profiles suggests EZH2 and JARID2 shift their binding ability from DNA to RNA in HepG2 cells to promote cancer development in HCC. Our study provided a comprehensive and distinct binding profile on RNAs and DNAs of EZH2 and JARID2 in liver cancer cell lines, suggesting their potential novel functional manners to promote HCC development.

## Introduction

HCC, among the top leading causes of cancer-related death, is one of the most common malignant tumors with a low survival and high morbidity rate worldwide (1, 2). Excessive drinking, cirrhosis, and hepatitis B/C virus infection are common exogenous factors of HCC (3) and can trigger genetic and epigenetic alterations in liver cells, one of the major molecular pathogenesis of HCC (4). Although genomics variations using genome-wide association study (GWAS) from hundreds of liver tumor samples have found tens of HCC associated mutations from coding and noncoding regions (5, 6), most of their functions were unknown and could not be widely used due to tumor heterogeneity. Epigenetic modifications and transcriptional regulation have emerged as novel and vital regulation factors during hepatic carcinogenesis (7). Integration analysis of multi-platform data of HCC gave us new perspectives of the molecular landscape (8). Decoding the relationship between epigenetic modifications and transcriptional regulation could substantially enhance our understanding of the pathogenesis of HCC.

EZH2 belongs to the polycomb group genes (PcGs) family, which is important for repressing transcription by epigenetic regulation. EZH2 can mediate gene silencing and regulate gene expression by trimethylation of Lys-27 in histone 3 (H3K27me3) (9). Several studies have shown the elevated expression level and cancer promotion effect of EZH2 in HCC, mainly through the polycomb repressive complex 2 (PRC2)-dependent roles as transcriptional repressors (10–13). Oncogenic mutations of EZH2 were also important for the regulation of cancer development, driving multiple layers changes within chromatin domains in cancer (14, 15). The target gene recruitment process of PRC2 has been illustrated by its cooperation with other molecules (16). The most well-studied interacting protein with PRC2 is JARID2 in embryonic stem cells (17–21). JARID2 belongs to Jumonji (Jmj) family proteins, which plays a stable PRC2 interactor in ES cells, HEK293 cells, HeLa cells, and mouse thymus. JARID2 is crucial in ES cell differentiation. It is involved in stopping the pluripotent network and triggering the expression of lineage-specific genes (19). JARID2 could modulate the methyltransferase activity of PRC2 by direct interaction (18, 22). The carcinogenic functions of JARID2 were also validated (23). JARID2 could promote the invasion and metastasis of HCC (24). It has also shown the connect role of lncRNA Meg3 in facilitating JARID2-PRC2 interaction on chromatin during differentiation of mouse ESCs (25), and the interaction between RNA and PRC2 can be reshaped with the presence of JARID2 (26). These results suggest the lncRNAs–JARID2–PRC2 trimeric complex could extensively regulate transcriptional profiling by altering epigenetic modifications. However, the functional mechanisms of this RNA–protein complex have not been extensively studied in cancer.

Long noncoding RNAs (lncRNAs), with lower expression levels and higher tissue specificity than mRNAs (27), can drive many important cancer phenotypes through their interactions with other cellular macromolecules, including DNA, protein, and RNA (28). Several lncRNAs, including Metastasis-Related Lung Adenocarcinoma Transcript 1 (MALAT1), H19 Imprinted Maternally Expressed Transcript (H19), and some others, have been shown to be candidate targets of promising therapeutic and diagnostic modalities for several cancers (28). H19 has been shown to associate with EZH2 and increase bladder cancer metastasis (29), or as an miRNA sponge to enhance the expression of oncogenes. But its functions in liver cancer are not well defined because of the contradictory evidence provided by different studies (30). Similar contradictory results can also be found for lncRNA MALAT1 (31). It suggests that although the regulatory roles of lncRNAs are important, elucidating their functional mechanisms in cancer needs further studies.

In this study, we comprehensively compared the RNA and DNA binding profiles of EZH2 and JARID2 between HepG2 and THLE-2 cell lines, respectively. By interacting with the transcriptome data from The Cancer Genome Atlas (TCGA), we found that the specifically bound transcripts of EZH2 and JARID2 in HepG2 cells were higher expressed in HCC samples by analyzing the RNA-binding profiling from CLIP-seq data, showing the H3K27-independent role of EZH2 and JARID2. The RNAs bound by EZH2/JARID2 were also closely associated with cancer pathways in HepG2 cells. By integrating the DNA binding profiles of EZH2, JARID2, and H3K27me3, we extended the H3K27-dependent function of EZH2 and JARID2 by repressing the expression of anti-tumor genes in HepG2 cells, while we did not find these phenomena in THLE2 cells.

## Results

### EZH2 and JARID2 have co-function in liver cancer cells with higher expression

**Figure 1:**
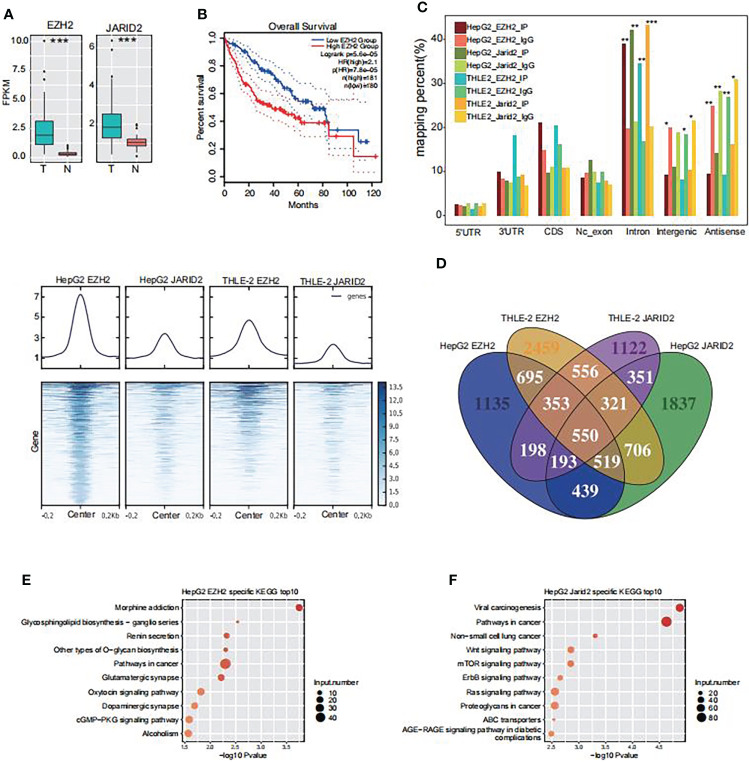
The RNA binding profiles of EZH2 and JARID2 in HepG2 and THLE-2 cell lines, respectively. **(A)** Box plot showing the transcriptional level of EZH2 and JARID2 in HCC tumor and adjacent normal samples from TCGA database. FPKM, Fragments Per Kilobase of exon model per Million mapped fragments. **(B)** Overall survival in Liver Hepatocellular Carcinoma patients with different expression levels of EZH2 and JARID2. **(C)** Heat map presentation of all eight samples reads density around the center of peaks from HepG2 JARID2 sample. **(D)** Venn diagram showing the overlapped genes among the bound genes by EZH2 and JARID2 in HepG2 and THLE-2 cell lines. **(E)** Bubble plot showing the top ten enriched KEGG pathways for genes specifically bound by EZH2 in HepG2 cells compared with THLE-2 cells. **(F)** Bubble plot showing the top ten enriched KEGG pathways for genes specifically bound by JARID2 in HepG2 cells compared with THLE-2 cells.

It has been reported that EZH2 and JARID2 are upregulated in human HCC (12, 24). To validate this phenomenon, we downloaded transcriptome data (RNA-seq) of 51 HCC and normal pairs from The Cancer Genome Atlas (TCGA) database. Principal component analysis showed the normal samples exhibited a more homogeneous profile than the tumor samples (**Figure S1A**), suggesting the heterogeneous nature of tumor tissue (32). From the TCGA data, we found EZH2 and JARID2 were both significantly upregulated in the HCC group (*p*-value <0.0001, **Figure 1A**) and well correlated with similar expression levels (R = 0.74, **Figure S1B**). Higher expression level of EZH2 and JARID2 both showed poorer prognosis of Liver Hepatocellular Carcinoma (LIHC) patients (**Figure 1B**, **Figure S1C**). We then checked the correlation of transcripts from the PRC2 complex, including SUZ12 Polycomb Repressive Complex 2 Subunit (SUZ12), Embryonic Ectoderm Development gene (EED), and Retinoblastoma protein associated protein 46/48 (RBAP46/48). We found their correlation coefficients were as high as those of EZH2 and JARID2 (**Figure S1C**). In addition to the correlation analysis, we observed that the expression level of SUZ12 and RBAP46/48 was much higher than EZH2 and JARID2 (**Figure S1D**), while EED and EZH2 showed lower expression levels. The incoordinate expression pattern between EZH2 and other PCR2 proteins suggests that EZH2 and JARID2 may have PRC2 complex independent roles in HCC.

To validate our hypothesis, we performed cross-linking and immunoprecipitation and then sequenced the pull-down RNAs (CLIP-seq) to investigate the binding profiles of EZH2 and JARID2 in liver cells. Two immortalized human liver cells were used in our study, including HepG2 and THLE-2. HepG2 and THLE-2 cells are both liver-derived but from cancerous and normal liver tissues, respectively. This experimental design enabled us to investigate the molecular pathogenesis of HCC. Two biological replicates were performed in parallel for each protein in each cell. After aligning the filtered reads to the human genome (GRCH38) by TopHat2 (33), sample correlation analysis revealed the biological replicates of IP samples were well correlated and clearly separated from IgG samples (**Figure S1E**), suggesting that the CLIP-seq experiment was successful. Because of the high correlation between two biological replicates, we merged the two aligned bam files into one to do the following analysis. The genomic distribution of the aligned reads showed that EZH2 and JARID2 both prefer bound intronic regions in both cell lines compared with IgG (*p*-value <0.01, Fisher’s exact test, **Figure 1B**). While the IgG samples were enriched in intergenic and antisense regions (**Figure 1B**), which can be treated as binding noise, the binding enrichment in intronic regions suggests that these two proteins may bind to precursor RNAs (pre-RNAs) and regulate the alternative splicing process.

We used a peak calling method to detect the bound regions of EZH2 and JARID2 in these two cell lines (34), and obtained 2,264–9,122 bound peaks from 1,620 to 3,932 bound genes (**Table S1**). The read distribution around the bound peaks from HepG2 EZH2 CLIP-seq showed that the binding density was also mildly accumulated in other IP samples (**Figure 1C**), and vice versa. The binding coordination was higher for samples of the same protein than from those of the same cell type (**Figure 1C**). These results demonstrated that EZH2 and JARID2 may share lots of overlapping binding peaks, suggesting their co-function in liver cancer cells.

### EZH2 and JARID2 tend to synergistically bind cancer-related genes in HepG2 cells

Previous studies suggest that EZH2 binding on RNAs is somewhat promiscuous (26, 35, 36). Overlapping analysis of these bound genes revealed that 4,530 (40.87%) and 1,936 (17.47%) genes were bound by two and three CLIP-seq samples, respectively (**Figure 1D**), suggesting the binding profiles of EZH2 and JARID2 were not so well correlated with each other in these two cells. To further decipher the functional influences of EZH2/JARID2 binding, we analyzed the enrichment functions of bound genes by using the Gene Ontology (GO) and Kyoto Encyclopedia of Genes and Genomes (KEGG) databases. To make a comparison between HepG2 and THLE-2 cells, we found specifically bound genes by EZH2 in HepG2 cells (1,965 genes) were significantly enriched in cancer related pathways, including those in cancer (**Figure 1D**). Specific genes bound by EZH2 in THLE-2 cells (3,042 genes) showed different pathways, including TNF signaling pathway, HTLV-1 infection, and other pathways (**Figure S1F**). Similar results were also observed for genes bound by JARID2 (**Figures 1E**, **F** and **Figure S1G**). These results suggest that the RNA binding profile of EZH2 and JARID2 showed cell specific character and they may play important roles in the carcinogenesis of liver by binding to cancer related transcripts.

### EZH2/JARID2 binding stabilizes the expression level of bound transcripts

**Figure 2:**
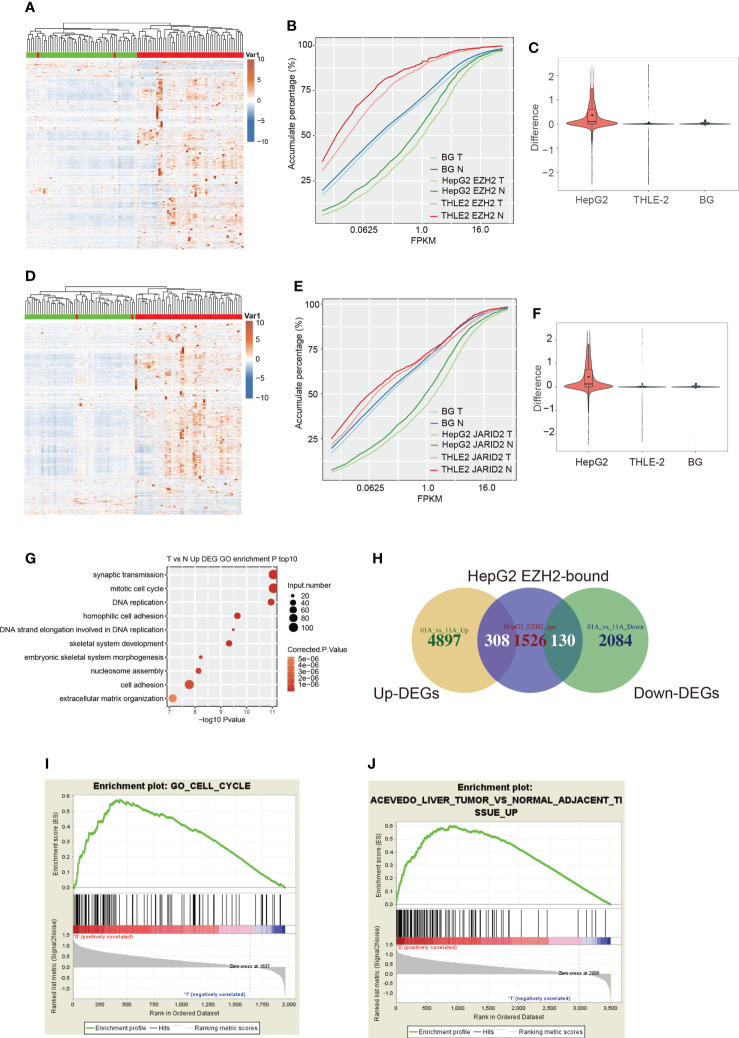
The expression pattern and functional analysis of EZH2 bound transcripts in HepG2 cells. **(A)** Hierarchical clustering heatmap showing the expression pattern of genes specifically bound by EZH2 in HepG2 cells compared with THLE-2 cells. The expression levels of genes were obtained from the TCGA database. The samples with the green bar were adjacent normal samples, and the samples with the red bar were HCC tumor samples. **(B)** Accumulative plot showing the expression pattern of specifically EZH2-bound genes in HepG2 or THLE-2 cells, as well as all the expressed genes. **(C)** Violin plot showing the difference value between tumor and normal samples for specifically EZH2-bound genes in HepG2 or THLE-2 cells, as well as all the expressed genes. **(D**–**F)** The three figures showing the similar results presented in **(A–C)** but for the bound genes by JARID2. **(G)** Bubble plot showing the top ten enriched biological processes for genes upregulated in HCC compared with adjacent normal samples. **(H)** Venn diagram showing the overlapped genes between EZH2 binding and DEGs from the TCGA database. **(I)** GSEA analysis results showing the enriched gene set for genes specifically bound by EZH2 in HepG2 cells compared with THLE-2 cells. **(J)** GSEA analysis results showing the enriched gene set for genes specifically bound by JARID2 in HepG2 cells compared with THLE-2 cells.

We then explored how EZH2 and JARID2 influence the bound transcripts in HepG2 and THLE-2 cells. Post-transcriptional regulation by RNA-binding proteins (RBPs) finally has an impact on the steady level and translation efficiency of transcripts (37, 38). We explored the transcriptional level of these bound genes from TCGA HCC samples. Most of the bound genes by EZH2 in HepG2 cells showed increased expression levels in HCC patients (**Figure 2A**). To eliminate the background influence of total expressed genes, we plotted the expression level of all expressed genes and found a slightly higher level in tumors than normal (**Figure S2A**). When adding the specific bound genes in THLE-2 cells and all expressed genes, we found that the specific bound genes in HepG2 cells were expressed much higher than all expressed and THLE-2 specific genes (*p*-value <2.2e−16, Kolmogorov–Smirnov (K–S) test, **Figure 2B**). The difference between tumor and normal was also more significant for EZH2 specifically bound genes in HepG2 cells than others (*p*-value = 0, *t*-test, **Figure 2C**). Similar results were also obtained for JARID2 (**Figures 2D–F**). We performed this analysis for the transcripts that were specifically bound by EZH2/JARID2 in THLE-2 cells. Although slightly higher expression levels were observed in tumor samples (**Figures S2B, C**), the expression levels of these transcripts were much lower than EZH2/JARID2 specifically bound genes in HepG2 cells (**Figures 2B, C, E, F**). These results indicated that EZH2/JARID2 had a significant preference to bind to the highly expressed and cancer-related genes in HCC. To further explore this finding, we obtained the differentially expressed genes (DEGs) in these 51 HCC patient pairs. Much more upregulated genes (5,233 up-DEGs and 2,220 down-DEGs) were found in the tumor compared with adjacent normal tissues (**Table S2**). The enriched GO biological process terms of the upregulated genes showed they were mainly enriched in cell cycle and DNA replication related terms (**Figure 2G**). The downregulated genes were mainly enriched in metabolic and inflammatory pathways (**Figure S2D**). Overlapping results between the DEGs and EZH2/JARID2 bound genes showed that they overlapped in the upregulated and downregulated gene sets (**Figure 2H**, **Figure S2E**). We then performed gene set enrichment analysis (GSEA) (39) to further study the functions of EZH2/JARID2 bound genes. Bound genes of EZH2 (HepG2 specific) were mainly enriched in cell cycle set (ES = 0.58, **Figure 2I**), consistent with the enriched functional terms of up-DEGs. Genes upregulated in liver tumors compared to the normal adjacent tissue were significantly enriched in bound genes of JARID2 (ES = 0.6, **Figure 2J**). Meanwhile, a small number of bound genes were also enriched in the set of genes downregulated in the liver tumor compared to the normal adjacent tissue for both EZH2 and JARID2 (**Figures S2F, G**). In summary, we demonstrated that EZH2/JARID2 has a significant and specific preference to bind to the highly expressed and cancer-related genes in the HepG2 cell line, which may partially explain the contribution of EZH2/JARID2 in the carcinogenesis and progression of HCC.

### Specific lncRNAs were bound by both EZH2 and JARID2 in HepG2 but not in THLE-2 cells

**Figure 3:**
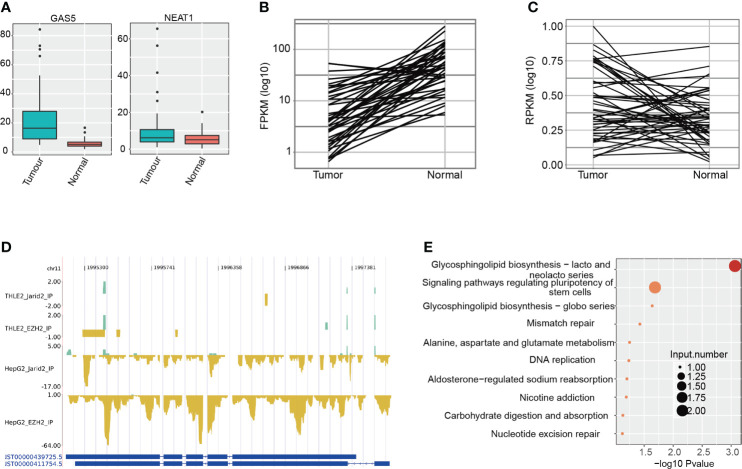
Analysis of lncRNAs bound by EZH2 and JARID2 in HepG2 and THLE-2 cell lines. **(A)** Boxplot showing the expression pattern of two lncRNAs bound by EZH2 and JARID2 in HepG2 and THLE-2 cell lines. The expression levels of lncRNAs were obtained from the TCGA database. **(B)** Line plot showing the expression pattern of lncRNA H19 bound by EZH2 and JARID2 specific for HepG2 cell line. The expression levels of lncRNAs were obtained from the TCGA database. **(C)** Line plot showing the expression pattern of lncRNA PDXDC2P bound by EZH2 and JARID2 specific for HepG2 cell line. The expression levels of lncRNAs were obtained from tumor-normal paired HCC samples in the TCGA database. **(D)** CLIP-seq reads density presentation for lncRNA H19 in the four CLIP-seq datasets. **(E)** Bubble plot showing the top ten KEGG pathways of the genes co-expressed with H19.

Previous studies have shown that lncRNA could drive many important cancer phenotypes by interacting with cellular macromolecules, including proteins (28). It is also reported that EZH2 and JARID2 regulate gene transcription by interacting with lncRNAs in ESCs and cancers (40). The highly bound intensity in intronic regions (**Figure 1B**) inspired us to determine whether EZH2 and JARID2 bind to lncRNAs to regulate gene transcription in HCC. We collected the lncRNAs bound by EZH2 and JARID2, with a total of 1,538 bound lncRNAs (**Table S3**). LncRNA Maternally Expressed 3 (MEG3), proven to participate in the chromatin organization of JARID2 and PRC2 in ESCs (25), was only weakly bound by JARID2 in THLE-2 cells (**Figure S3A**). We identified 13 lncRNAs with binding signals in all the four CLIP-seq samples, including Nuclear Enriched Abundant Transcript 1 (NEAT1), Small Nucleolar RNA Host Gene 1 (SNHG1), growth arrestspecific 5 (GAS5), and LINC00910 (**Table S3**). NEAT1 and GAS5 have been reported to positively promote HCC development by an anti-apoptosis effect (41, 42), and we have proved that NEAT1 and GAS5 have been bound by EZH2 (**Figure S3**). Expression levels of NEAT1 and GAS5 were also significantly increased in tumor samples compared with adjacent normal tissue (*p*-value = 2.16e−8 for GAS5 and *p*-value = 0.01 for NEAT1, *t*-test, **Figure 3A**), suggesting that they inhibit apoptosis in HCC, perhaps by interacting with EZH2 and JARID2. Besides, we performed Meanwhile, we also detected seven lncRNAs specifically bound by EZH2 and JARID2 in HepG2 cells (**Table S3**), including Colon Cancer Associated Transcript 1 (CCAT1), RP11-13J10.1, H19, Putative Pyridoxal-dependent Decarboxylase Domain-containing Protein 2 (PDXDC2P), CTA-109P11.4, CTB-12A17.2, and RP11-775H9.2. By interacting with the TCGA RNA-seq data, we found expression levels of H19 and PDXDC2P were significantly changed between tumor and adjacent normal (*p*-value = 9.39e−8 for H19 and *p*-value = 0.0083 for PDXDC2P, *t*-test, **Figures 3B, C**). The decreased expression of H19 in tumors was interesting. Its biological functions in different cancers were not concordant and even contradictory evidences were emerged in HCC studies (30). We found that H19 was specifically bound by EZH2 and JARID2 in hepatoma cell lines and repressed in HCC patients (**Figure 3D**). To identify the interaction specificity between EZH2/JARID2 and lncRNAs, we re-analyzed the CLIP-seq of EZH2 in mouse ESC (17, 25), and the CLIP-seq of EZH2 in colon cancer HCT-116 cells (40). However, no binding signal was found in these datasets (data not shown), suggesting the binding specificity in liver cancer cells. We then analyzed the co-expressed genes with H19 to explore its functions in HCC, and obtained 151 positively correlated genes. Functional enriched KEGG pathways of these co-expressed genes included glycosphingolipid biosynthesis, signaling pathways regulating pluripotency of stem cells, and DNA replication (**Figure 3E**), consistent with their known functions in embryonic and cancer cells. However, the opposite expression between H19 and its interacted protein EZH2/JARID2 indicated that EZH2/JARID2 perhaps promote HCC development by destabilizing the transcript level of H19.

### EZH2 binds DNA in a PRC2-independent manner in HepG2 cells

**Figure 4:**
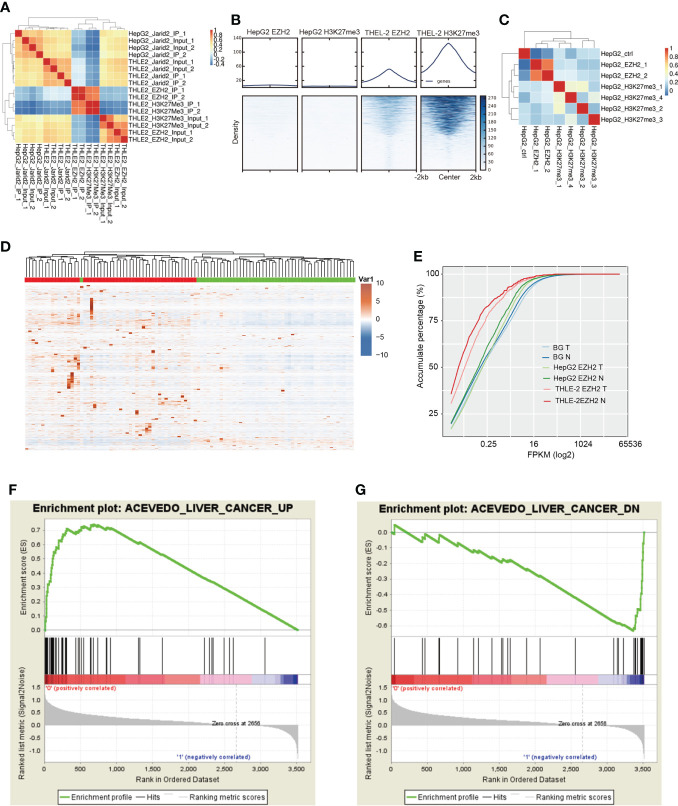
ChIP-seq results showing that EZH2 could increase the expression level of bound genes. **(A)** Hierarchical clustering sample correlation showing that EZH2 and H3K27me3 binding profile were highly correlated in THLE-2 cells. **(B)** Heatmap presentation of ChIP-seq samples reads density around the center of peaks from THLE-2 EZH2 and H3K27me3 samples. **(C)** Hierarchical clustering sample correlation showing that EZH2 and H3K27me3 binding profile were lowly correlated in HepG2 cells. **(D)** Hierarchical clustering heatmap showing the expression pattern of genes specifically bound by EZH2 in HepG2 cells compared with THLE-2 cells. The expression levels of genes were obtained from the TCGA database. The samples with the green bar were adjacent normal samples, and the samples with the red bar were HCC tumor samples. **(E)** Accumulative plot showing the expression pattern of specifically EZH2-bound genes in HepG2 or THLE-2 cells from ChIP-seq results, as well as all the expressed genes. **(F)** GSEA analysis result showing the enriched gene set for top ranked genes specifically bound by JARID2 in HepG2 cells compared with THLE-2 cells. **(G)** GSEA analysis results showing the enriched gene set for bottom-ranked genes specifically bound by JARID2 in HepG2 cells compared with THLE-2 cells.

A previous study has shown that intronic RNAs bound by EZH2 could regulate endogenous gene expression by directing chromatin complexes toward their genomic loci (40). We also identified the enriched intronic binding feature of EZH2 and JARID2 (**Figure 1B**). We then performed chromatin immunoprecipitation and sequencing (ChIP-seq) experiments to decipher the epigenetic influence on HCC. Two biological replicates were performed in parallel. The ChIP-seq data of EZH2 and H3K27me3 for HepG2 cells were downloaded from The Encyclopedia of DNA Elements (ENCODE) (43). From the global sample correlation analysis, in THLE-2 cells, the bound density of EZH2 and H3K27me3 was well correlated (**Figure 4A**), indicating the canonical H3K27 methyltransferase function of EZH2. However, the correlation between JARID2 and EZH2 DNA binding profiles was negative (**Figure 4A**), which was not consistent with their co-functioning manner of regulating development in ESCs. Reads density around peak center analysis also revealed the high consistency of EZH2 and H3K27me3 in THLE-2 cells, while very weak signals were detected in HepG2 cells (**Figure 4B**), suggesting that the EZH2 and H3K27me3 profiles were dramatically changed in liver cancer cells. From **Figure 4B** and **Figure S4A**, we can also detect that the DNA binding ability of EZH2 was much weaker than that of THLE-2 cells. For the downloaded HepG2 ChIP-seq data, the correlation between EZH2 and H3K27me3 was also much lower than that in THLE-2 cells (**Figure 4C**). The read density around EZH2 bound peaks in HepG2 cells showed weak signal for the H3K27me3 marker (**Figures S4A, B**), suggesting that the DNA binding profile of EZH2 in HepG2 cell lines is dramatically altered with attenuated H3K27 tri-methylation function. It has been reported that the repression function of EZH2 by H3K27-independent mechanisms in HCC (10).

To validate our hypothesis, we analyzed the expression levels and functions of EZH2 bound genes specifically identified in HepG2 cells. We found most of these genes showed significantly elevated expression levels in tumor samples compared with adjacent normal (*p*-value = 5.38e−6, K–S test, **Figures 4D, E**), and the tumor and normal samples were clearly separated (**Figure 4D**), indicating that EZH2 could increase the transcriptional level of bound genes. For the specifically EZH2-bound genes in the THLE-2 cell line, although their difference between tumor and normal was significant (*p*-value = 0.02, K–S test), the expression level was much lower than the all-expressed and HepG2-specific EZH2-bound genes (**Figure 4E**). These results indicated that the methyltransferase role of EZH2 was inhibited in liver cancer cells. We then analyzed the functions of the bound genes to further understand EZH2 function in HepG2 cells. The Gene Set Enrichment Analysis (GSEA) results showed that many liver cancer upregulated genes were significantly enriched at the top rank of these genes (*p*-value = 0, **Figure 4F**). A few liver cancer downregulated genes were also observed at the bottom rank of these genes (p-value = 0, **Figure 4G**). The unexpected result suggests that EZH2 has clear functions to promote or repress the up or downexpressed genes in HCC, respectively, and then promote the development of HCC.

### EZH2 presents high binding to RNAs and weak binding to DNA in HCC

**Figure 5:**
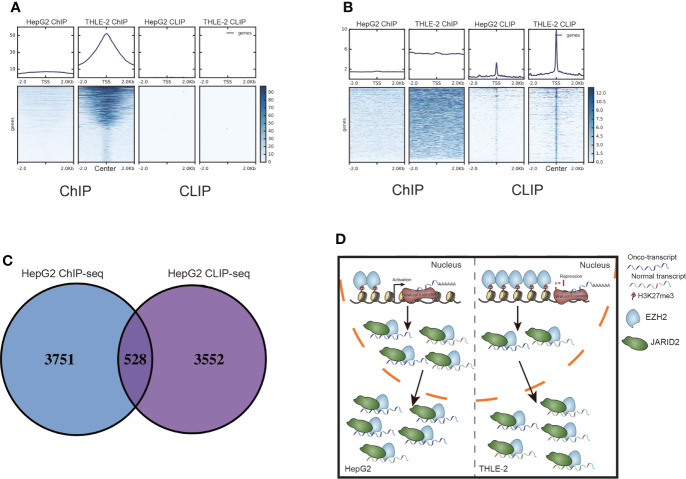
The interaction between bound RNAs and DNAs by EZH2 and JARID2. **(A)** Heatmap presentation for the reads density of ChIP-seq and CLIP-seq samples around the center of peaks from THLE-2 EZH2 DNA binding profile (ChIP-seq). **(B)** Heatmap presentation for the reads density of ChIP-seq and CLIP-seq samples around the center of peaks from THLE-2 EZH2 RNA binding profile (CLIP-seq). **(C)** Venn diagram showing the bound genes by EZH2 ChIP-seq and CLIP-seq samples from HepG2 cells. **(D)** Working model of EZH2 and JARID2 in HCC. In HepG2 cells, EZH2 exhibits weak DNA binding, thus activating more onco-expression and then binding more to highly expressed RNAs together with JARID2 to produce a more mature onco-transcript. In THLE-2 cells, the DNA binding ability of EZH2 is maintained and represses the expression of oncogenes, and then reduced binding with RNA to produce a less mature onco-transcript.

Based on the above discoveries, we further investigated the interaction between RNA binding and DNA binding of these two proteins. It has been reported that RNAs were enriched at the PRC2 target genes for RNA-mediated regulation (44). We investigated the RNA binding density around the DNA binding site of EZH2. The result showed no protruding signal was found for RNAs around the DNA binding peak (**Figure 5A**), and vice versa (**Figure 5B**). This phenomenon was also detected in HepG2 cells (**Figures S4A, B**). Overlapping analysis between bound transcripts and bound genes also showed no significant enrichment (*p*-value = 1, Hypergeometric test, **Figure 5C**). These results suggested that the binding feature between RNA and DNA was independent for EZH2. We then compared the binding profile difference between HepG2 and THLE-2 cells. As shown in **Figures 4A, C**, higher co-occurrence between EZH2 and H3K27me3 was found in THLE-2 cells than HepG2 cells. As highly expressed RNAs shuttle PRC2 away from chromatin (45). We proposed that the binding profile of EZH2 were globally changed between HepG2 and THLE-2 cells. In HepG2 cells, EZH2 prefers binding to highly expressed RNAs in HCC and exhibits weak DNA binding and methylation ability. On the contrary, its DNA binding ability is maintained and represses the expression of bound genes in THLE-2 cells. In this case, few EZH2 proteins have the opportunity to bind to RNAs (**Figure 5D**). The shifted binding profile of EZH2 between HepG2 and THLE-2 may play important roles during the initiation and development of HCC.

## Discussion

Molecular mechanisms of HCC pathogenesis and development have been extensively studied due to their therapeutic importance in the future (46). Integration of multidimensional high throughput data from cancer cell lines improved our understanding of the molecular features that contribute to cancer phenotypes (47). In this study, we explored the nucleic acid binding profiles of two carcinogenic proteins, EZH2 and JARID2, and their potential epigenetic and post-transcriptional regulation of gene expression in two different liver cell lines. By exploring the binding profiles of RNAs of EZH2 and HepG2, we found they could efficiently bind highly expressed transcripts in HCC samples. The DNA binding profile and interacted analysis with TCGA HCC RNA-seq data also showed the dramatically altered DNA binding profile of EZH2 between HepG2 and THLE-2 cell lines. These results extend our understanding of the carcinogenic functions of EZH2 and JARID2 in HCC.

As the catalytic component and interacted partner of PRC2, EZH2, and JARID2 were most well known as transcription repressor to regulate the differentiation and development of embryonic cells (16, 48). It is also reported EZH2 could interact with RNAs to exert its functions dependent or independent on PRC2, especially in cancers (17, 40). Despite that EZH2 could promote HCC development by repressing the transcription of some anti-tumor miRNAs and genes (10, 11), the global regulatory mechanisms of EZH2 in HCC were poorly understood. The finding that EZH2 and JARID2 prefer to bind intronic regions of transcripts indicates they may regulate the progress of primary RNAs or they may bind intronic RNAs to regulate epigenetic targets (40). We also detected the higher expression level of specifically bound transcripts in HepG2 cells, indicating that EZH2 and JARID2 may stabilize the expression level of bound transcripts. Cell-type specific binding profiles of EZH2 and JARID2 indicate their distinct functions in different cell lines or tissues, which are also supported by the binding profile of Suz12, another component of PRC2 (49), and these functions are perhaps independent of PRC2.

In this study, we found that the DNA binding profile between EZH2 and H3K27me3 was lower correlated in HepG2 cells than in THLE-2 cells. The transcription activation role of EZH2 has been proposed in cancer (50, 51). In HepG2 cells, we found EZH2 binds to the genes highly expressed in HCC patients, including those genes participating in the cancer pathway, indicating that EZH2 performs transcription activation roles in HCC by interacting with other proteins. The low correlation between EZH2 and H3K27me3 and the repressed binding signal on DNA for EZH2 and H3K27me3 in HepG2 suggests the DNA binding ability of EZH2 was attenuated in liver cancer. To the contrary, EZH2 prefers to bind highly expressed transcripts in liver cancer, indicating many more EZH2 molecules interact with RNAs rather than DNAs.

It is also reported that PRC2-binding RNA motifs are enriched at PRC2-binding sites on chromatin and H3K27me3-modified nucleosomes in embryonic cells (44). By interacting the RNA and DNA binding profiles in HepG2 cell line, we found in liver cell lines, it may be two independent progresses that EZH2 binds RNAs and DNAs separately, although the binding targets are with similar carcinogenesis functions. *In vitro* studies have shown that RNA and DNA binding to PRC2 are mutually exclusive (45). We also did not observe the close relationship between DNA and RNA-binding features of EZH2. These results suggest that EZH2 could independently bind RNAs and DNAs at the same time but could stabilize the bound-RNA level or promote the transcription of bound-DNAs. At the same time, it needs further and deeper studies to explore the intrinsic relationship between bound RNAs and DNAs in cancer cells or patients.

We also extensively analyzed the bound lncRNAs by EZH2 and JARID2. Some lncRNAs showed cell-type-specific binding features. Most interestingly, H19 lncRNA with a cell-type-specific binding feature showed reverse transcription level with EZH2 and JARID2. Recent study showed TGF-β and H19 axis *via* Sox2 importantly regulates hepatocarcinogenesis (52), regardless its lower expression level in HCC tumor tissue. Cholangiocyte-derived exosomal-H19 plays a critical role in cholestatic liver injury (53). H19 also increases bladder cancer metastasis by associating with EZH2 and inhibiting E-cadherin expression (29). The contradictory between H19 expression and functions in HCC may be explained that H19 functions as a hepatocarcinogenesis role at the initiatory stage of HCC but is then repressed by other factors, which depends on its context within the process of tumor progression (54). Functional analysis revealed that H19 may regulate the biogenesis of glycosphingolipids, inhibition of which could serve as an antitumor method (55, 56). Its association with EZH2 and JARID2 may also influence the molecular functions of EZH2 and EZH2/JARID2, perhaps promoting HCC development by destabilizing the transcript level of H19 (57).

Finally, we need to think about the clinical roles of EZH2 and JARID2 in HCC patients. As we have detected, there is definitely a significant change in RNA and DNA binding, especially in RNA and HCC-related genes with higher expression. Thus, these two proteins may be detected as two kinds of biomarkers to predict the development of HCC and be applied in early diagnosis and prognosis prediction, which will benefit more HCC patients and improve their treatment outcomes.

## Conclusions

In summary, we proposed a model in which EZH2 and JARID2 shift their RNA and DNA binding profiles between liver cancer and normal cell lines. Stronger RNA binding ability and weaker DNA binding signal in tumor cells suggest that the canonical methyltransferase functions of EZH2 may be repressed in liver cancer cells, implying their novel regulatory mechanisms of EZH2 and JARID2 in promoting HCC occurrence and development.

## Methods

### Cell culture of HepG2 and THLE-2 cell

HepG2 (85011430, Sigma, USA) was cultured with RPMI1640 (ThermoFisher, USA) supplemented with 10% FbS (10099141C, Gibco, USA), 100 U ml^−1^ penicillin/streptomycin (15140122, Gibco, USA) and 4 mM glutamine (glutamine, Gibco, USA). THLE-2 Cell (CRL-2706,ATCC, US) was cultured with the BEGM Bullet Kit (CC-3170, Lonza, USA). All the cell lines were cultured in 24-well plates and stored in a cell incubator (51032124, Thermo Fisher Scientific, MA, USA), growth conditions: 37°C, 5% CO2.

### CLIP-seq methods

The HepG2 and THLE-2 cells (~10^7^) were cross-linked on ice with UV irradiation type C (254 nm) at 400 mJ/cm^2^ in the presence of cold PBS (4 ml per 15-cm dish). Cells were lysed in cold wash buffer (1× PBS, 0.1% SDS, 0.5% NP-40, and 0.5% sodium deoxycholate) supplemented with a 200 U/ml RNase inhibitor (Takara) and protease inhibitor cocktail (Roche) and treated with RQ I RNase-Free DNase (promega, 1 U/μl) to prevent DNA contamination, followed by partial digestion with MNase (Thermo) to further release the protein-unprotected RNA fragments. The protein–RNA complex was immunoprecipitated by incubating with DynaBeads protein A conjugated with anti-EZH2 antibody, or anti-JARID2 antibody, IgG at 4 °C for 2 h. The DynaBeads were sequentially washed with lysis buffer, high-salt buffer (250 mM Tris 7.4, 750 mM NaCl, 10 mM EDTA, 0.1% SDS, 0.5% NP-40, and 0.5 deoxycholate), and PNK buffer (50 mM Tris, 20 mM EGTA, and 0.5% NP-40) for two times, respectively. RNA was dephosphorylated at the 3’ end and phosphorylated at the 5’ end. The protein-RNA complex was separated by a 4%–12% NuPAGE Bis-Tris gel (Nvirogen) and the region of the gel 30 kDa above the protein size was excised. Protein was digested by proteinase K and RNA was isolated by TRIzol (Invitrogen).

### ChIP-seq method

Cells (~10^7^) from the same batch with CLIP-seq were cross-linked by 1% formaldehyde and stopped the reaction by 0.125 M glycine. The cross-linked cells were lysed in RIPA buffer(50 mM Tris 7.4, 150 mM NaCl, 2 mM EDTA, 0.1% SDS, 0.5% NP-40, and 0.5% deoxycholate) and sonicated to generate DNA fragments of 200–500 bp. Protein–DNA complexes were immunoprecipitated by incubating with ChIP-grade Protein A/G Magnetic Beads conjugated with anti-EZH2 antibody, or anti-JARID2 antibody, IgG at 4 °C for 2 h. The beads were sequentially washed with LiCl IP Wash Buffer (100 mM Tris (PH 7.5), 500 mM LiCl, 1% NP-40, and 0.5 deoxycholate) five times, TE buffer [10 Mm Tris (PH 8.0), 1 mM EDTA (PH 8.0)] for one time, and Resuspend sample with 100ul Elution Buffer (100 mM NaHCO3, 1% SDS) and reverse cross-linked by overnight incubation at 65 °C. After sequential RNase A and proteinase K treatment, DNA fragments were purified by phenol extraction and ethanol precipitation. To generate libraries with the ThruPLEX^®^ DNA-seq Kit (R400427, Rubicon Genomics) according to the instructions of the manufacturer.

For high-throughput sequencing, the CLIP-seq and ChIP-seq libraries were prepared following the instructions of the manufacturer (Gnomegen) and applied to the Illumina Hiseq 2000 system for 100 nt pair-end sequencing by ABLife Inc. (Wuhan, China).

### CLIP-seq data analysis

For CLIP-seq data, adaptors and low quality bases were trimmed from raw sequencing reads using Cutadapt (Version 1.7.1) software (58) with default parameters, and reads <16 nt were discarded. After quality filtering, we merged the biological replicates and aligned the combined reads to the human-GRCH38 genome using TopHat2 (33) with no more than four mismatches. After reads were aligned onto the genome, we discarded reads with multiple genomic locations due to their ambiguous origination. Identically aligned reads were counted and merged as unique reads. To globally predict the binding sites of EZH2 and JARID2, we used the “ABLIRC” program (34) to extract positive binding sites and discard negative binding sites from IP samples compared with IgG samples (p-value <0.05).

### ChIP-seq data analysis

For ChIP-seq data, quality filtering criteria were the same as for CLIP-seq data. After quality filtering, we aligned the filtered reads to the human-GRCH38 genome using Bowtie2 (59) with no more than two mismatches. The quality of ChIP-seq data were then assessed by the standard criteria of the ENCODE consortia (60). The fraction of reads falling within peak regions is measured by the FRiP (fraction of reads in peaks) method. The irreproducible discovery rate (IDR) analysis methodology was used to assess the replicate agreement. The normalized ratio between the fragment-length cross correlation peak and the background cross-correlation (normalized strand coefficient, NSC) and the ratio between the fragment length peak and the read-length peak (relative strand correlation, RSC) were used to assess the signal-to-noise ratios in a ChIP-seq experiment. To identify the binding sites of EZH2, JARID2, and H3K27me3, we used Model-based Analysis for ChIP-seq (MACS) version 1.4 (61) to obtain the binding sites. The input samples without immunoprecipitation were treated as background. DeepTools (62) were used for the assignment of genomic features such as relative location to transcription start sites (TSSs) to the peak centers and visualization of binding profiles.

We also downloaded the public ChIP-seq data of EZH2 and H3K27me3 in HepG2 cells from the ENCODE project (43), and analyzed these datasets with the analysis pipeline.

### RNA-seq data analysis

We downloaded the 102 transcriptome expression data of 51 HCC patients (tumor and normal pairs) from the TCGA database (https://www.cancer.gov/about-nci/organization/ccg/research/structural-genomics/tcga), including the read number and normalized expression level (fragments per kilobase per million, FPKM) files for all the expressed genes. We performed differentially expressed genes (DEGs) analysis using the edgeR (63) package from R software. Genes with FDR <0.05 and |log2 Fold Change| >1 were selected as DEGs.

### Functional enrichment analysis of gene sets

For selected gene sets, Gene Ontology (GO) and KEGG enrichment analysis was performed with KOBAS 2.0 (37). A hypergeometric test was performed with robust FDR correction to obtain an adjusted P-value between certain tested gene groups and genes annotated in the reference genome. A Gene Set Enrichment Analysis (GSEA) (39) was carried out to analyze the genes specifically bound by JARID2 in HepG2 cells compared with THLE-2 cells. The number of permutations was set to 1,000, and the metric for ranking genes was set as Signal2Noise, and a false discovery rate (FDR) of <25% was recognized as statistically significant.

### Statistical analysis

Significant *p*-values of expression differences were calculated by either Student’s *t*-test when only two groups were compared, or hypergeometric test for Venn diagram and functional term enrichment analysis. Fisher’s exact test was used to calculate the genomic region enrichment. The Kolmogorov–Smirnov test (K–S test) was used to calculate the difference in the cumulative curve for bound genes.

## Data availability statement

The datasets presented in this study can be found in online repositories. The names of the repository/repositories and accession number(s) can be found below: https://www.ncbi.nlm.nih.gov/GSE133984.

## Ethics statement

Ethical review and approval was not required for the study on human participants in accordance with the local legislation and institutional requirements. Written informed consent from the patients/participants or patients/participants legal guardian/next of kin was not required to participate in this study in accordance with the national legislation and the institutional requirements.

## Author contributions

Conception: ZW, YF, and LL. Design of the work: FS, and GX. Experiments: MZ, YX, YL, and FL. Interpretation of data: KH, YuZ, XH, YaW, WZ, YJX, ZX, YeW, XD, and JX. Draft the work: ZW, MZ, and KH. Substantively revised the work: LL. All authors contributed to the article and approved the submitted version.

## Funding

This work was supported by the National Key Research and Development Program of China [2017YFA0205200 to LL]; the National Natural Science Foundation of China [81641110 to GX, 81571785 to LL, 81771957 to LL, 81502642 to MZ, 81801811 to LL, 81901857 to LL, and 81660166 to ZW]; the Natural Science Foundation Guangdong Province, China [2015A030313725 to GX, 2016A030311055 to LL, 2016A030313770 to LL, and 2018A030313074 to YF]; the Science and Technology Program of Guangzhou, China [201707010305 to MZ]; the Medical Research Foundation of Guangdong Province, China [A2017427 to LL]; the Youth Science Foundation of Guangdong Second Provincial General Hospital [YQ2016-001 to KH], and the Academic Leaders Project of Major Disciplines in Jiangxi Province [20182BCB22018 to ZW]. The funding bodies did not participate in the design of the study and collection, analysis, and interpretation of data, and in writing the manuscript.

## Acknowledgments

We thank the staffs from ABLife Inc., Wuhan for their discussion about the manuscript.

## Conflict of interest

Authors YuZ and YQX were employed by ABLife Inc.

The remaining authors declare that the research was conducted in the absence of any commercial or financial relationships that could be construed as a potential conflict of interest

## Publisher’s note

All claims expressed in this article are solely those of the authors and do not necessarily represent those of their affiliated organizations, or those of the publisher, the editors and the reviewers. Any product that may be evaluated in this article, or claim that may be made by its manufacturer, is not guaranteed or endorsed by the publisher.
