# Supplementary material for: Distinct binding pattern of EZH2 and JARID2 on RNAs and DNAs in hepatocellular carcinoma development
Source: Front Oncol. 2022 Dec 9;12:904633. doi: 10.3389/fonc.2022.904633 (PMC9792092; doi:10.3389/fonc.2022.904633)
Supplement: Supplementary file 4 [file DataSheet_1.docx]

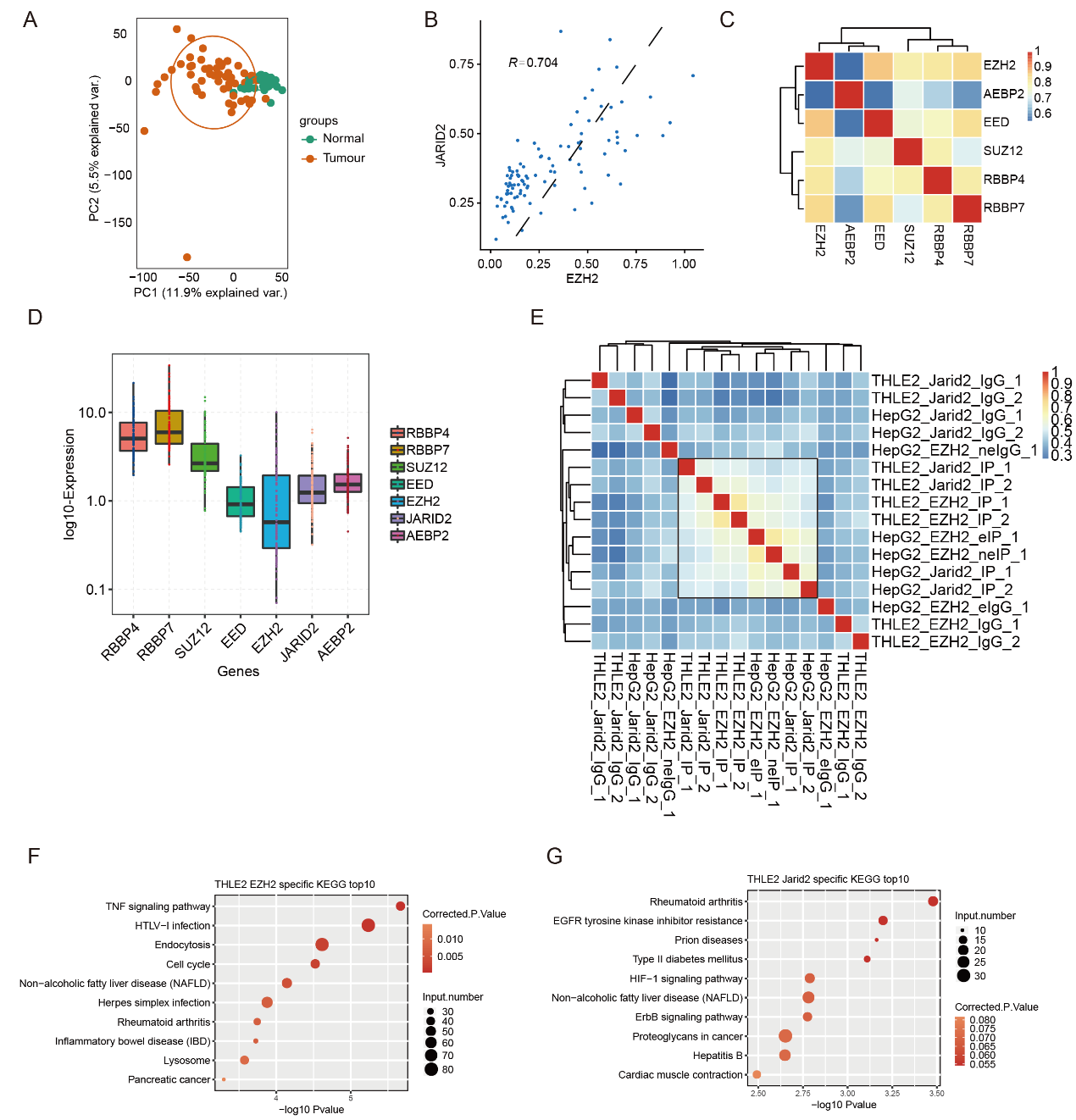


**Figure S1.** **The RNA binding profiles were well correlated between EZH2 and JARID2 in HepG2 and THLE-2 cell lines, respectively. (A)** PCA analysis showing the global distribution of HCC tumor and adjacent normal samples from TCGA database. (**B**) Dot plot showing the expression correlation between JARID2 and EZH2 in HCC samples. (**C**) Heat map clustering showing the correlation among genes in PRC2 complex and interacted gene AEBP2. (**D**) Boxplot showing the expression level of genes in PRC2 complex and interacted gene AEBP2. (**E**) Heat map clustering showing the sample correlation of eight CLIP-seq samples from HepG2 and THLE-2 cell lines. (**F**) Bubble plot showing the top ten enriched KEGG pathways for genes specifically bound by EZH2 in THLE-2 cells compared with HepG2 cells. (**G**) Bubble plot showing the top ten enriched KEGG pathways for genes specifically bound by JARID2 in THLE-2 cells compared with HepG2 cells.


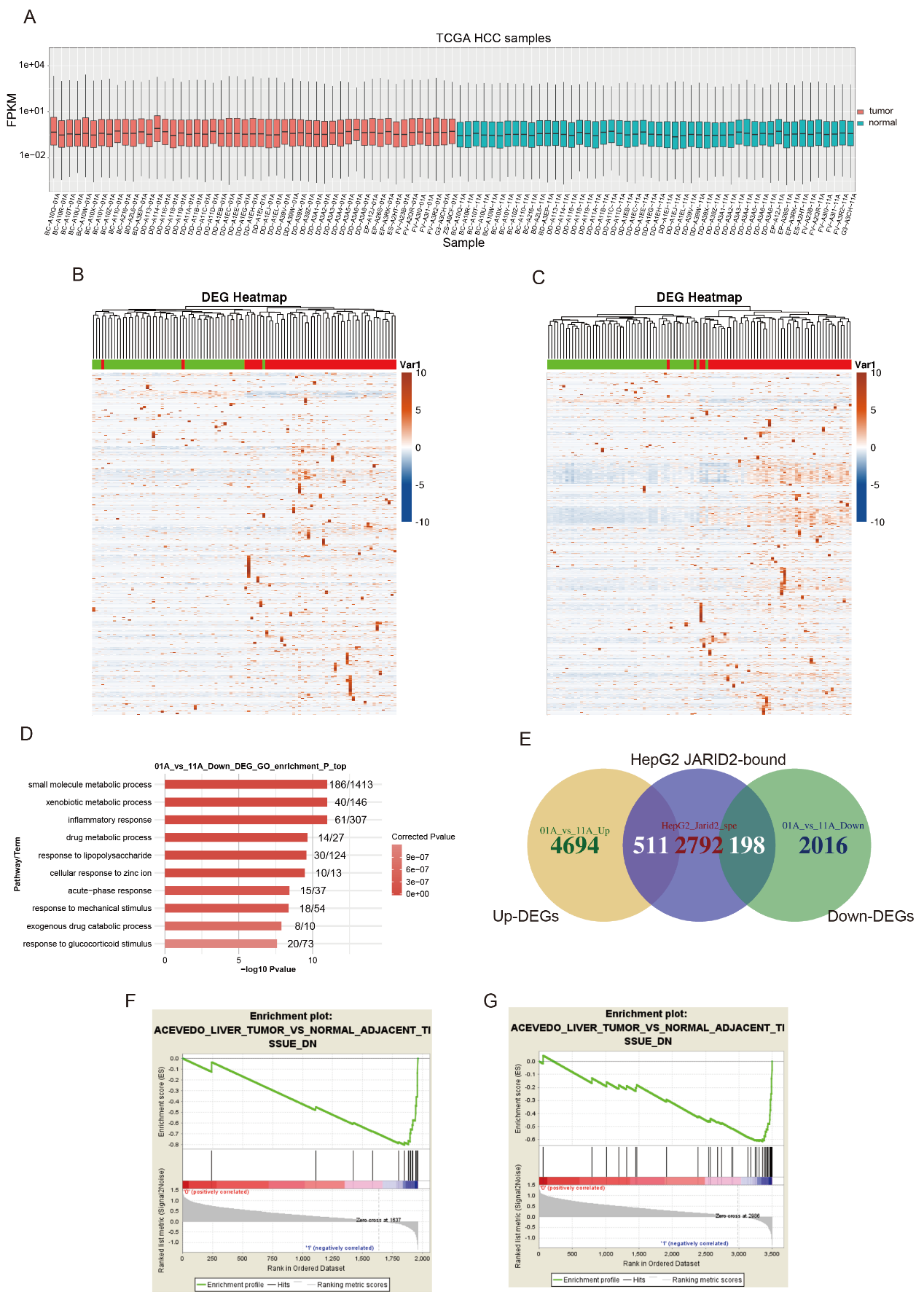


**Figure S2. The expression pattern and functional analysis of EZH2 bound transcripts in THLE-2 cells.** (**A**) Box plot showing the global expression level of all 102 HCC tumor and adjacent normal samples from TCGA database. Orange represents tumor samples, and blue for normal samples. (**B**) Hierarchical clustering heatmap showing the expression pattern of genes specifically bound by EZH2 in THLE-2 cells compared with JARID2 cells. The expression levels of genes were obtained from TCGA database. The samples with green bar were adjacent normal samples, and the samples with red bar were HCC tumor samples. (**C**) Hierarchical clustering heatmap showing the expression pattern of genes specifically bound by JARID2 in THLE-2 cells compared with JARID2 cells. The expression levels of genes were obtained from TCGA database. The samples with green bar were adjacent normal samples, and the samples with red bar were HCC tumor samples. (**D**) Bubble plot showing the top ten enriched biological processes for genes down-regulated in HCC compared with adjacent normal samples. (**E**) Venn diagram showing the overlapped genes between JARID2 binding and DEGs from TCGA database. (**F**) GSEA analysis result showing the enriched gene set for genes specifically bound by EZH2 in HepG2 cells compared with THLE-2 cells. (**G**) GSEA analysis result showing the enriched gene set for genes specifically bound by JARID2 in HepG2 cells compared with THLE-2 cells.


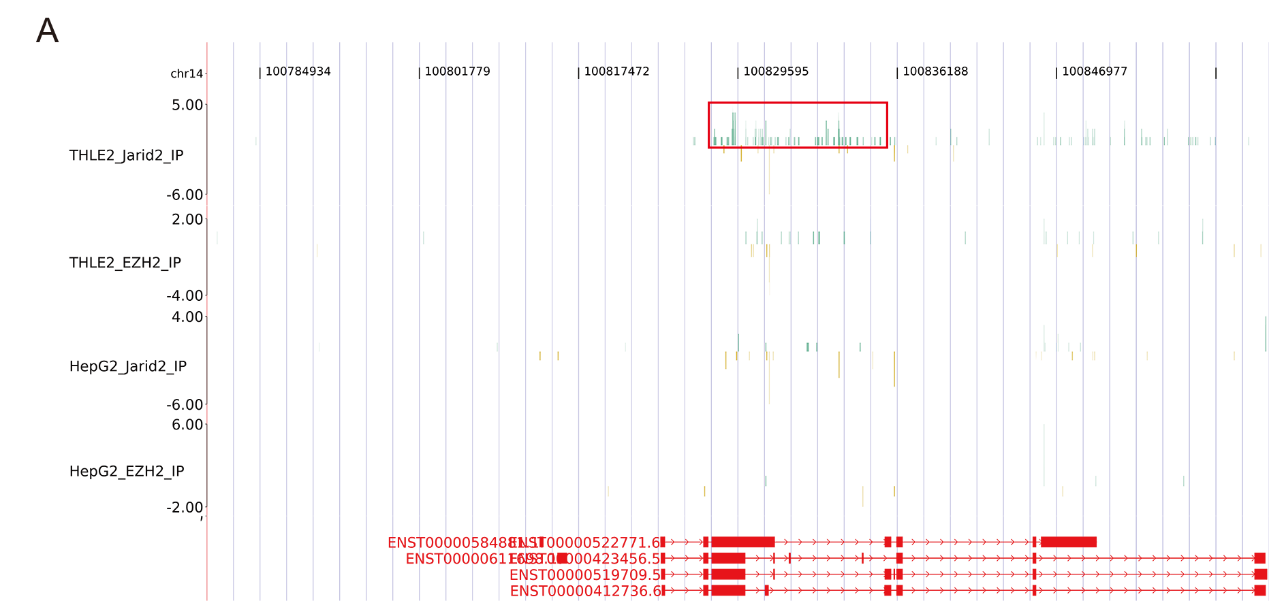


**Figure S3. (A) CLIP-seq reads density presentation for lncRNA MEG3 in the four CLIP-seq datasets.** Red rectangular frame represented the binding signals for JARID2 in THLE-2 cells.


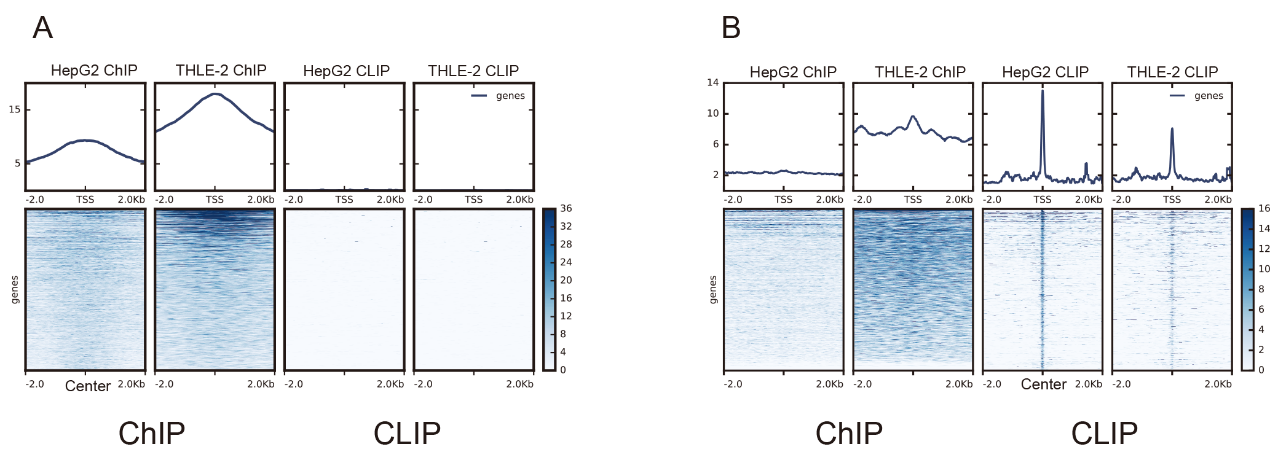


**Figure S4. The interaction between bound RNAs and DNAs by EZH2.** (A) Heatmap presentation for the reads density of ChIP-seq and CLIP-seq samples around the center of peaks from HepG2 EZH2 DNA binding profile (ChIP-seq). (B) Heatmap presentation for the reads density of ChIP-seq and CLIP-seq samples around the center of peaks from HepG2 EZH2 RNA binding profile (CLIP-seq).
